# Bayesian kinetic modeling for tracer-based metabolomic data

**DOI:** 10.1186/s12859-023-05211-5

**Published:** 2023-03-22

**Authors:** Xu Zhang, Ya Su, Andrew N. Lane, Arnold J. Stromberg, Teresa W. M. Fan, Chi Wang

**Affiliations:** 1 Dr. Bing Zhang Department of Statistics, University of Kentucky, Lexington, 40536 USA; 2 Department of Statistical Sciences and Operations Research, Virginia Commonwealth University, Richmond, 23220 USA; 3 Markey Cancer Center, University of Kentucky, Lexington, 40536 USA; 4 Center for Environmental and Systems Biochemistry, University of Kentucky, Lexington, 40536 USA; 5 Department of Toxicology and Cancer Biology, University of Kentucky, Lexington, 40536 USA; 6 Division of Cancer Biostatistics, Department of Internal Medicine, University of Kentucky, Lexington, 40536 USA

**Keywords:** SIRM, Kinetic modeling, Bayesian method

## Abstract

**Background:**

Stable Isotope Resolved Metabolomics (SIRM) is a new biological approach that uses stable isotope tracers such as uniformly $^{13}C$-enriched glucose ($^{13}C_6$-Glc) to trace metabolic pathways or networks at the atomic level in complex biological systems. Non-steady-state kinetic modeling based on SIRM data uses sets of simultaneous ordinary differential equations (ODEs) to quantitatively characterize the dynamic behavior of metabolic networks. It has been increasingly used to understand the regulation of normal metabolism and dysregulation in the development of diseases. However, fitting a kinetic model is challenging because there are usually multiple sets of parameter values that fit the data equally well, especially for large-scale kinetic models. In addition, there is a lack of statistically rigorous methods to compare kinetic model parameters between different experimental groups.

**Results:**

We propose a new Bayesian statistical framework to enhance parameter estimation and hypothesis testing for non-steady-state kinetic modeling of SIRM data. For estimating kinetic model parameters, we leverage the prior distribution not only to allow incorporation of experts’ knowledge but also to provide robust parameter estimation. We also introduce a shrinkage approach for borrowing information across the ensemble of metabolites to stably estimate the variance of an individual isotopomer. In addition, we use a component-wise adaptive Metropolis algorithm with delayed rejection to perform efficient Monte Carlo sampling of the posterior distribution over high-dimensional parameter space. For comparing kinetic model parameters between experimental groups, we propose a new reparameterization method that converts the complex hypothesis testing problem into a more tractable parameter estimation problem. We also propose an inference procedure based on credible interval and credible value. Our method is freely available for academic use at https://github.com/xuzhang0131/MCMCFlux.

**Conclusions:**

Our new Bayesian framework provides robust estimation of kinetic model parameters and enables rigorous comparison of model parameters between experimental groups. Simulation studies and application to a lung cancer study demonstrate that our framework performs well for non-steady-state kinetic modeling of SIRM data.

**Supplementary Information:**

The online version contains supplementary material available at 10.1186/s12859-023-05211-5.

## Background

The metabolome comprises the full set of compounds and the biochemical reactions that represent the functional phenotypes of living organisms. It changes in response to either internal environments (such as mutations or alterations in gene/protein expression) or external perturbations (such as altered nutrient supply) which occur during disease progression. Unraveling the dynamics of changes in the metabolome is a key to elucidating molecular regulations at both gene and protein levels that underlie macroscopic phenotypes such as individual variations in disease development or drug susceptibility. In recent years, a powerful approach, Stable Isotope Resolved Metabolomics (SIRM) [1–11] has been developed to decipher metabolic networks and changes in disease states, which can be linked to altered regulation at gene and protein expression levels. Based on mass spectrometry and nuclear magnetic resonance techniques, SIRM uses stable isotope tracers such as uniformly $^{13}\text {C}$-enriched glucose ($^{13}\text {C}_\text {6}\text {-Glc}$) to trace metabolic pathways or networks at the atomic level [1, 7, 9, 11–16] in cells, tissues, living organisms, or even human patients, that respond to disease development or drug treatment. By determining the atomic position (isotopomers) of stable isotope incorporation into relevant metabolites, the enzymes that are involved in the altered biochemical conversion of parent tracers into these metabolites can be systematically identified along with their possible allosteric regulator(s). In cancer research, for example, SIRM studies have uncovered key reprogrammed metabolic events and regulations in cancer cells and tissues [14, 17–23].

Kinetic modeling analysis is a commonly used approach to quantitatively model metabolic pathway dynamics based on total metabolite profiles and/or tracer data. Many kinetic modeling analyses [24–26] focus on the case that the system has achieved both metabolic and isotopic steady state. Several statistical and machine learning algorithms have been developed to characterize kinetic models and estimate model parameters for such models, see Saa and Nielsen [27] and Cuperlovic-Culf [28] for reviews. However, the steady state is difficult to achieve in complex biological systems such as mammalian cells, tissues, or in whole bodies [29]. An important development is the extension of kinetic modeling analysis to non-metabolic and non-isotopic steady flux analysis based on ordinary differential equations (ODEs) that represent the kinetics of individual or groups of enzyme-catalyzed reactions within the proposed metabolic networks [30–35]. Non-steady-state kinetic models are more realistic and have been increasingly used to understand the regulation of normal metabolism, the development of disease, and to predict metabolic reactions upon genetic and environmental perturbation [36–39]. Such characterization is then necessary for the development of novel therapeutic strategies.

For statistical analyses of non-steady-state kinetic modeling, current methods [31, 40, 41] are weighted least squares-based, which seek an optimal set of parameters to minimize the difference between model and the observed data. However, due to the complexity of the model and limited number of replicates, there are usually multiple sets of parameter values that fit the data equally well [40]. Without imposing additional constraints, it is difficult to unambiguously determine all model parameters. In addition, quantifying the uncertainty in parameter estimation is also challenging because the Hessian matrix, which is required to calculate the standard error of parameter estimator, is often ill-conditioned. Further, without an accurate characterization of the standard error of parameter estimator, it is difficult to statistically rigorously compare kinetic model parameters as well as metabolic flux between different experimental groups, e.g. treatment versus control. Consequently, Fan *et al.* [41] only compared the point estimate of a parameter between groups without providing a quantification of statistical significance such as a p-value.

Here we describe a new Bayesian statistical framework for non-steady-state kinetic modeling of SIRM data to provide accurate and robust estimation of model parameters and to allow rigorous comparison of model parameters between experimental groups. For estimation of kinetic model parameters, our Bayesian method incorporates experts’ knowledge about the most likely region of model parameters by a prior distribution, which yields robust estimates of parameters and avoids retrieving scientifically unrealistic parameter values. In addition, we propose a Bayesian shrinkage prior for the variances of the isotopomer abundances to stabilize their estimation, and thus more reliably quantify the uncertainty in kinetic model parameter estimation for limited sample sizes. Further, we introduce advanced Markov chain Monte Carlo (MCMC) methods including adaptive Metropolis [42–44] and delayed rejection [45, 46] to improve the efficiency of posterior sampling under the high-dimensional situation. For comparison of kinetic parameters between experimental groups, we propose a novel reparameterization method that converts the complex hypothesis testing problem, which involves high-dimensional integrals, into a more tractable parameter estimation problem. Based on the posterior sample of that parameter, a credible interval can be easily constructed to assess the null hypothesis. We further propose a *credible value* to quantify the likelihood for the null hypothesis to hold. Simulation studies were conducted to evaluate the accuracy of the proposed method in estimating kinetic model parameters and detecting differentiated parameters between experimental groups. By applying our method, we characterized the dysregulation of purine synthesis in lung squamous cell carcinoma tissues.

## Methods

In this section, we introduce a kinetic model to characterize the dynamics of biochemical processes. Based on this model, we propose a Bayesian framework to estimate the model parameters and conduct hypothesis tests comparing parameter values between experimental groups.

### A Bayesian kinetic model

Biochemical processes can be described by systems of biochemical reactions, showing how the reactants are transformed and related to the products. The most common approach to model the evolution of such a system over time is dynamical system modeling via ODEs. These ODEs characterize the functional relationship between reactants at a specific time point *t*, where the time derivatives quantify the biochemical reaction rates between reactants. Specifically, let $\varvec{y}_{tj} = (y_{tj1}, \ldots , y_{tjn})'$ be a vector of observed concentrations of *n* isotopomers at time *t* in independent repeated sample *j*, $j=1,\ldots ,m$, and $\varvec{\mu }_{t}$ be a vector of mean concentrations of isotopomers at time t. We consider the following model

1 $$\begin{aligned} \log \varvec{y}_{tj}= & {} \log \varvec{\mu }_t + \varvec{\delta }_{tj}, \nonumber \\ \frac{d\varvec{\mu }_t}{dt}= & {} \varvec{f}(\varvec{\mu }_t;\varvec{\beta },t), \end{aligned}$$

where $\varvec{f}$ is the formula of a set of pre-specified nonlinear ODEs with respect to $\varvec{\mu }_t$ for different isotopomers, $\varvec{\beta }$ is a vector of unknown ODE model parameters on the logarithmic scale, and $\varvec{\delta }_{tj}$ is a vector of independent random error terms. The ODE model parameters represent the rate constants of the biochemical reactions which are determined by the enzyme parameters, such as the $K_m$ (the Michaelis-Menten constant) and $k_{cat}$ (the turnover number, the number of times each enzyme site converts substrate to product per unit time), which are our main parameters of interest. Note that because ODE parameters are always positive, we consider $\varvec{\beta }$, the natural logarithm of ODE parameters, for the convenience of prior specification and posterior sampling. The error term $\varvec{\delta }_{tj}$ describes the technical and biological variations. It has been suggested that a normally distributed additive error term on the logarithm of the data is appropriate when modeling biochemical reactions [47]. Therefore, we assume $\varvec{\delta }_{tj} \sim N(0,\Sigma _\delta )$, where $\Sigma _\delta$ is a diagonal matrix with the *i*th diagonal element being $\sigma _i^2$. Let $\varvec{\sigma }^2$ be a vector of the $\sigma _i^2$’s.

Although our interest lies in $\varvec{\beta }$, the inference relies heavily on a stable estimate of $\varvec{\sigma }^2$. When we assume discrete *T* time points are observed, the likelihood function of the model, $L(\varvec{\beta }, \varvec{\sigma }^2)$, is

$$\begin{aligned} |\Sigma _\delta |^{-\frac{mT}{2}}\exp \bigg \{-\frac{1}{2}\sum _t^T \sum _{j=1}^m(\log \varvec{y}_{tj}-\log \varvec{\mu }_t)^{'}\Sigma _\delta ^{-1}(\log \varvec{y}_{tj}-\log \varvec{\mu }_t)\bigg \}, \end{aligned}$$

where $\varvec{\mu }_t$ is a function of $\varvec{\beta }$ and *t* defined in Eq. (1), and $\Sigma _\delta$ contains $\varvec{\sigma }^2$. This model is highly nonlinear and the parameters are embedded within the ODEs. It is challenging to use the maximum likelihood method directly to obtain model parameter estimations since taking derivatives with respect to the parameters within ODEs is not trivial, though it can be done numerically, or explicitly for known function forms such as Hill equation or the Michaelis-Menten equation). Bayesian methods are valuable alternatives to obtain posterior samples of parameters without the need to take derivatives. They also provide a natural and principled way to combine the observed data with experts’ prior knowledge and information about the parameters, which is the key to achieve robust parameter estimation for a complex system with a limited sample size. Let $p(\varvec{\beta })$ and $p(\varvec{\sigma }^2)$ be priors of $\varvec{\beta }$ and $\varvec{\sigma }^2$, respectively, and assume that they are independent with each other, the joint posterior distribution of $\varvec{\beta }$ and $\varvec{\sigma }^2$ is

$$\begin{aligned} p(\varvec{\beta }, \varvec{\sigma }^2 | \varvec{y}) \propto L(\varvec{\beta }, \varvec{\sigma }^2)p(\varvec{\beta }) p(\varvec{\sigma }^2), \end{aligned}$$

where $\varvec{y} = (\varvec{y}_{tj})$ is the observed data across time point $t=1,\ldots , T$ and replicate $j=1,\ldots , m$.

### Prior specification

We assume that the prior distribution of $\varvec{\beta }$ is a truncated multivariate normal distribution. Specifically, we first consider a multivariate normal distribution with mean $\varvec{\xi }$ and covariance matrix $\Lambda$, where values of $\varvec{\xi }$ and $\Lambda$ are specified based on experts’ knowledge and using literature data and databases such as BRENDA [48]. We then further constrain the distribution within a reasonable value range between $\varvec{\zeta }_l$ and $\varvec{\zeta }_u$, which are also specified by experimentalists, to rule out some extreme values that are not biologically possible, and thus enhances the robustness of our method under the high-dimensional situation. Note that we do not require the experts to specify very narrow ranges. The ranges are determined by the amount of information available about each enzyme. If there is extensive information about an enzyme under different conditions, a relatively narrow range will be specified, whereas for less well characterized enzymes, a wider range will be used.

When only a limited number of replicates are collected for an individual isotopomer, it is unlikely to provide a stable estimate of $\sigma _i^2$ without any regulation. We consider a Bayesian shrinkage approach that assumes a common prior for all $\sigma _i^2$’s, which regulates the variance estimate by borrowing information from the ensemble of isotopomers. This approach has been shown to provide a more stable estimate of the variance in transcriptomics studies [49]. Importantly, a stable estimate of the variance can also enhance the estimation of $\varvec{\beta }$. Specifically, we assume the common prior is an inverse gamma distribution with shape parameter $\alpha _*$ and scale parameter $\kappa _*$. The hyperparameters $\alpha _*$ and $\kappa _*$ are empirically determined based on the sample variances of all isotopomers, i.e.

$$\begin{aligned} \begin{aligned} \alpha _*&= \bigg (\frac{1}{nT}\sum _{t=1}^{T}\sum _{i=1}^{n}s_{it}^2\bigg )^2\bigg /\bigg \{\frac{1}{nT-1} \sum _{t=1}^T\sum _{i=1}^{n}(s_{it}^2-\bar{s^2})^2\bigg \} + 2, \\ \kappa _*&= \bigg (\frac{1}{nT}\sum _{t=1}^{T}\sum _{i=1}^{n}s_{it}^2\bigg )(\alpha _*-1), \end{aligned} \end{aligned}$$

where $s_{it}^2 = \{\sum _{j=1}^{m}(\log y_{tji}-\sum _{j=1}^{m} \log y_{tji}/m)^2\}/(m-1)$ denotes the sample variance over *m* replicates for each isotopomer *i* at time point *t*, $\bar{s^2} = (\sum _{t=1}^T\sum _{i=1}^{n}s_{it}^2)/(nT)$ stands for the average of all sample variances across time and isotopomer.

To demonstrate how the priors impact parameter estimation, consider the logarithm of the posterior

$$\begin{aligned} \begin{aligned} \log p(\varvec{\beta }, \varvec{\sigma }^2| \varvec{y}) \propto&- mT \log |\Sigma _\delta |- \sum _t^T\sum _j^m(\log \varvec{y}_{tj}-\log \varvec{\mu }_t)^{'}\Sigma _\delta ^{-1}\\&(\log \varvec{y}_{tj}-\log \varvec{\mu }_t)-(\varvec{\beta }-\varvec{\xi })^{'}\Lambda ^{-1}(\varvec{\beta }-\varvec{\xi })\\&+ 2 \sum _i^n \bigg \{(-\alpha _*-1)\log \sigma _i^2-\frac{\kappa _*}{\sigma _i^2}\bigg \}. \end{aligned} \end{aligned}$$

The above formula is a penalized log-likelihood function from a frequentist point of view, where a penalty term $-(\varvec{\beta }-\varvec{\xi })^{'}\Lambda ^{-1}(\varvec{\beta }-\varvec{\xi })$ is introduced to penalize $\varvec{\beta }$ value that is far away from its prior mean $\varvec{\xi }$. Likewise, a penalty term $(-\alpha _*-1)\log \sigma _i^2-\kappa _* / \sigma _i^2$ is to penalize $\sigma _i^2$ values far away from the overall empirical mean. Therefore, by using the prior distribution to regularize the parameter estimation, our method reduces the chances of getting extreme or implausible estimated values for those parameters.

### Inferring posterior distribution

Point estimates and credible intervals for model parameters are obtained from the posterior distribution using MCMC methods. We use the Gibbs sampling [50] to iteratively sample each element of $\varvec{\beta }$ and $\varvec{\sigma }^2$ from its conditional posterior distribution given all other parameters. In the next two subsections, we describe methods to sample from those conditional posterior distributions.

#### Posterior sampling for kinetic parameters

The conditional posterior sample of a kinetic parameter is obtained by using MCMC because that posterior has no analytic expression due to the complexity of the ODEs. Since the dimension of parameters is high, the standard Metropolis-Hastings algorithm is inefficient and may yield low rates of acceptance and poor mixing of the chain [44]. To improve efficiency, we opted to implement advanced MCMC methods including component-wise adaptive Metropolis [42–44] and delayed rejection [45, 46].

The adaptive Metropolis [43] tunes the covariance matrix of the proposal distribution in the Metropolis-Hastings algorithm based on the past sample path of the chain and automatically adapts the proposal distribution towards the target distribution. Based on the adaptive Metropolis algorithm, our proposal distribution for the *i*th element of the parameter vector, $\beta _i$, at the $(l+1)^{th}$ iteration is

$$\begin{aligned} q(\beta _i^* | \beta _i^l) = N(\beta _i^l, D_i^l), \end{aligned}$$

where the variance of the proposal distribution is

$$\begin{aligned} D_i^l = C_{ii}^l - C_{i,-i}^l{C_{-i-i}^l}^{-1}C_{-i,i}^l, \end{aligned}$$

where $C_{ii}^l$ is the (*i*, *i*) element of $C^l$, $C_{i,-i}^l$ is the $i^{th}$ row of $C^l$ after removing the $i^{th}$ column, $C_{-i,i}^l$ is the $i^{th}$ column of $C^l$ after removing the $i^{th}$ row, and $C_{-i-i}^l$ is the submatrix of $C^l$ leaving out the $i^{th}$ row and the $i^{th}$ column with

$$\begin{aligned} C^l = {\left\{ \begin{array}{ll} C_0, &{} l \le l_0, \\ s_dcov(\varvec{\beta }^1,\ldots ,\varvec{\beta }^l) + s_d\epsilon I_d, &{} l > l_0. \end{array}\right. } \end{aligned}$$

The matrix $C^l$ is fixed at $C_0$ for the first $l_0$ iterations and adaptive to the covariance of the past sample $\varvec{\beta }^1,\ldots ,\varvec{\beta }^l$ afterwards. The specifications of $C_0$, $s_d$ and $\epsilon$ are provided in Additional file 1.

The delayed rejection [45, 46] modifies the standard Metropolis-Hastings algorithm by delaying the rejection of proposed moves to improve MCMC efficiency in the Peskun sense [51]. When a candidate generated from the proposal distribution is rejected in Metropolis-Hastings, instead of advancing time and retaining the same position, a second candidate move is proposed with the acceptance probability adjusted to preserve the reversibility of the Markov chain relative to the target distribution. Specifically, when the proposed $\beta _i^*$ is rejected, we will do a further Metropolis step with scaled covariance, i.e.

$$\begin{aligned} q'(\beta _i^{**} | \beta _i^l, \beta _i^*) = N(\beta _i^l,\gamma D_i^l) \end{aligned}$$

where $\gamma$ is the scale parameter for delayed rejection. A detailed description of the component-wise adaptive Metropolis algorithm with delayed rejection is provided in Additional file 1.

#### Posterior sampling for error variances

The prior of $\sigma _i^2$, an inverse gamma distribution with shape parameter $\alpha _*$ and scale parameter $\kappa _*$, is a conjugate prior based on model (1). In Additional file 1, we show that the conditional posterior distribution of $\sigma _i^2$ given $\varvec{\beta }$ is an inverse gamma distribution with shape parameter $\alpha _*+ mT/2$ and scale parameter $\kappa _* +\sum _{t=1}^T\sum _{j=1}^{m}(\log y_{tji} - \log \mu _{ti})^2/2$, where $\mu _{ti}$ is the underlying concentration corresponding to the ODE in model (1) given the current value of $\varvec{\beta }$. The posterior sample of $\sigma _i^2$ is directly obtained from this distribution.

### Comparison of kinetic model parameters between experimental groups

A primary goal of many metabolomic studies is to identify metabolic alterations in response to disease development or drug treatment by comparing kinetic model parameters between two experimental groups, e.g. cancer vs. normal or drug-treated vs. control. Let $\varvec{\beta }^{(1)}$ and $\varvec{\beta }^{(2)}$ be kinetic parameters in two experimental groups, respectively. We focus on the situation of assessing whether the value of a specific kinetic model parameter, e.g. the $k^{th}$ parameter, is equal between experimental groups. Our hypothesis testing problem is:

$$\begin{aligned} H_0: \beta _k^{(1)} = \beta _k^{(2)} \text { vs. } H_1: \beta _k^{(1)} \ne \beta _k^{(2)}. \end{aligned}$$

A standard Bayesian hypothesis testing requires calculation of the following Bayes factor,

$$\begin{aligned} \frac{\int \dots \int p(\log \varvec{y} |\varvec{\beta }_{-k}^{(1)},\varvec{\beta }_{-k}^{(2)}, \beta _k^*)p(\beta _k^*, \varvec{\beta }_{-k}^{(1)},\varvec{\beta }_{-k}^{(2)})d\beta _k^* d\varvec{\beta }_{-k}^{(1)}d\varvec{\beta }_{-k}^{(2)}}{\int \dots \int p(\log \varvec{y} |\varvec{\beta }^{(1)},\varvec{\beta }^{(2)})p(\varvec{\beta }^{(1)},\varvec{\beta }^{(2)}) d\varvec{\beta }^{(1)}d\varvec{\beta }^{(2)}}, \end{aligned}$$

where $\varvec{\beta }_{-k}^{(1)}$ and $\varvec{\beta }_{-k}^{(2)}$ are vectors of parameters in $\varvec{\beta }^{(1)}$ and $\varvec{\beta }^{(2)}$ excluding $\beta _{k}^{(1)}$ and $\beta _{k}^{(2)}$, respectively, and $\beta _k^*$ denotes the common value of $\beta _k^{(1)}$ and $\beta _k^{(2)}$ under $H_0$. The Bayes factor involves intimidatingly high dimensional integrals that are very difficult to compute with a reasonable accuracy. To address the hypothesis testing problem while circumventing the high-dimensional integration, we propose the following reparameterization:

$$\begin{aligned} \eta _k = \beta _k^{(1)} - \beta _k^{(2)}. \end{aligned}$$

The hypothesis testing problem then becomes

$$\begin{aligned} H_0: \eta _k =0 \text { vs. } H_1: \eta _k \ne 0. \end{aligned}$$

Importantly, this hypothesis testing can be converted into a parameter estimation problem, where we first estimate $\eta _k$ by obtaining its posterior distribution, and then check the likelihood of $\eta _k = 0$ by constructing a credible interval and further calculating the tail probability under its posterior distribution.

Specifically, we jointly model data from the two experimental groups with the reparameterization in effect:

$$\begin{aligned} \log \varvec{y}_{tj}^{(1)}= & {} \log \varvec{\mu }_t^{(1)} + \varvec{\delta }_{tj}^{(1)},\\ \log \varvec{y}_{tj}^{(2)}= & {} \log \varvec{\mu }_t^{(2)} + \varvec{\delta }_{tj}^{(2)},\\ \frac{d\varvec{\mu }_t^{(1)}}{dt}= & {} f(\varvec{\mu }_t^{(1)}; \varvec{\beta }_{(-k)}^{(1)},\beta _k^{(1)},t),\\ \frac{d\varvec{\mu }_t^{(2)}}{dt}= & {} f(\varvec{\mu }_t^{(2)}; \varvec{\beta }_{(-k)}^{(2)},\beta _k^{(1)},\eta _k, t), \end{aligned}$$

where variables/parameters are defined similarly as before with an additional superscript to indicate the experimental group. We assume that $\varvec{\delta }_{tj}^{(1)}$ and $\varvec{\delta }_{tj}^{(2)}$ are independent and their *i*th elements follow normal distributions with mean 0 and variance $\sigma ^{2}_{i,(1)}$ and $\sigma ^2_{i,(2)}$, respectively.

The prior of $\varvec{\beta }^{(1)}$ and $\varvec{\beta }_{-k}^{(2)}$ are specified based on the expert’s knowledge and the prior of each $\sigma ^2_{i,(1)}$, $\sigma ^2_{i,(2)}$ is specified by an inverse gamma distribution using the shrinkage approach. The prior of $\eta _k$ is specified as a normal distribution with mean zero and variance equal to the variance of $\beta _k^{(1)}$. With some simple modification of the MCMC algorithm proposed in the last subsection, we obtain posterior samples of parameters $(\varvec{\beta }_{-k}^{(1)}, \varvec{\beta }_{-k}^{(2)}, \beta _k^{(1)},\eta _k, \varvec{\sigma }^{2}_{(1)}, \varvec{\sigma }^{2}_{(2)})$ with an additional sampling step for $\eta _k$. We then perform the hypothesis testing based on the posterior credible interval of $\eta _k$, where the two specific kinetic model parameters are considered as different if the 95% credible interval of $\eta _k$ does not cover 0. To further quantify the level of evidence supporting the null hypothesis, we investigate the highest level of credible interval that does not cover 0 and define a *credible value*

$$\begin{aligned} p = 1 - \max _{0 \le \alpha \le 1,0 \notin HDI_{\alpha }}\alpha , \end{aligned}$$

where $HDI_{\alpha }$ denotes the highest density interval of level $\alpha$ under the posterior distribution of $\eta _k$. This credible value reflects how extreme the null value is with respect to our posterior belief about $\eta _k$. In other words, it can be used as an empirical rule regarding how likely the null hypothesis value is correct. A similar idea has recently been investigated in simpler models in clinical trial studies [52]. As we will show in the simulation studies subsection, the distribution of the above credible values is concentrated around zero when the true values for $\beta _k^{(1)}$ and $\beta _k^{(2)}$ are different, while it is much more scattered between zero and one when the true values for $\beta _k^{(1)}$ and $\beta _k^{(2)}$ are the same. Thus, the credible value has a similar behavior as the frequentist’s p-value. We consider a credible value $<0.05$ as statistically significant. As the credible value is calculated based on $HDI_{\alpha }$ that can reflect deviation of $\eta _k$ from zero in either positive or negative direction, our test is a two-sided test.

The proposed algorithm could be applied in other scenarios (e.g. whether there is a difference in metabolic flux between groups) when the object of interest concerns the comparison of multiple kinetic parameters, more details are given in the Discussion section.

## Results

### Real data analysis

**Fig. 1:**
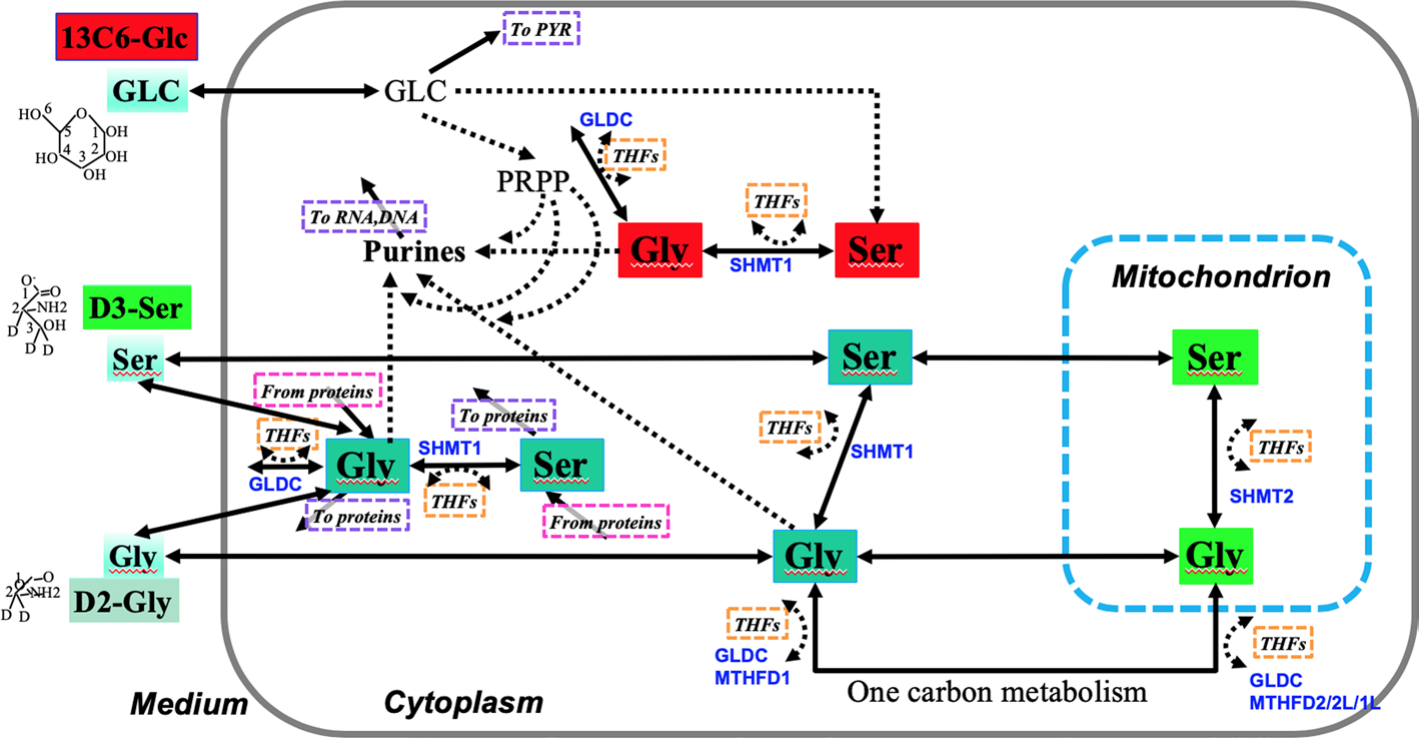
Tracer labeled three-pool purine synthesis process

We validated our proposed Bayesian framework by using published data in [41]. Fan *et al.* [41] employed multiplexed SIRM (i.e. multiple labeled substrates in the same experiment) to track the metabolism of $^{13}C_6$-Glc, $D_2$-glycine, $^{13}C_2$-glycine, and $D_3$-serine into purine nucleotides in thin slices of cancerous and matched noncancerous lung tissues freshly resected from a patient with lung squamous cell carcinoma. The data include abundances of isotopologues of serine and glycine and purine nucleotides in cancer and non-cancer tissue slices of that patient with three replicates, in both the tissue and the medium. Kinetic models were constructed and a weighted least square-based method was used to estimate model parameters. Fan *et al.* [41] considered a model with three distinct pools of cytoplasmic Ser/Gly (Fig. 1), where one pool is derived from $^{13}C_6$-Glc, the second is from exogenous $D_3$-Ser or $D_2$-Gly, and the third is from unlabeled sources. However, no statistical confidence interval or p-value was available due to the limitation of the weighted least squares-based method used in that paper. In this subsection, we use our proposed methods to reanalyze the data and provide more rigorous statistical inference. Initial ranges of kinetic parameters (Additional File 2: Table S1) were taken from literature sources for the appropriate human isoforms [48, 53].

#### Kinetic parameter estimation

**Fig. 2:**
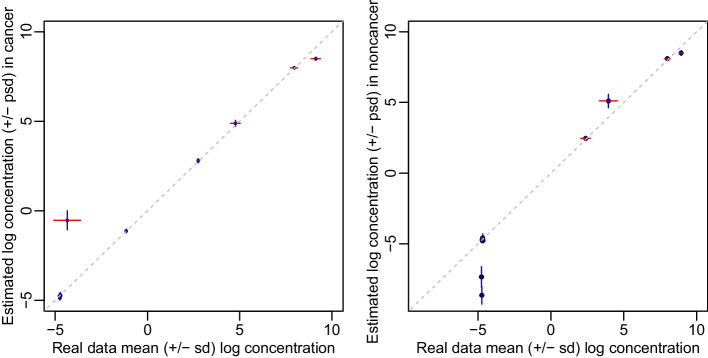
Comparison of estimated and observed isotopologue concentrations in log scale in lung cancer (left panel) and non-cancer (right panel) tissues. A red horizontal line indicates the interval “mean log concentration ± standard deviation (sd)” of observed concentrations for an isotopologue. A blue vertical line indicates the interval “estimated log concentrations ± posterior standard deviation (psd)” of the corresponding concentration. The gray dashed line is $y=x$

Following our proposed method, we obtained the posterior samples for all the $\beta$ parameters in model (1) based on 5500 MCMC iterations after 7500 burn-in iterations. The convergence of the MCMC chains was examined by the Geweke test (Additional File 2: Table S2). By inserting the $\beta$ values into model (1), we estimated the 24-hour concentrations of isotopologues. Fig. 2 compares those estimated log concentrations ± posterior standard deviations with mean log concentration ± standard deviation in the observed data for cancer and non-cancer groups, respectively. The estimated concentrations were very close to the observed ones for most isotopologues except for some low abundance ones. Likewise, the variations under the proposed model were also comparable to those presented by the observed data for most isotopologues except for some low abundance ones. These results indicate that our estimators perform well.

**Fig. 3:**
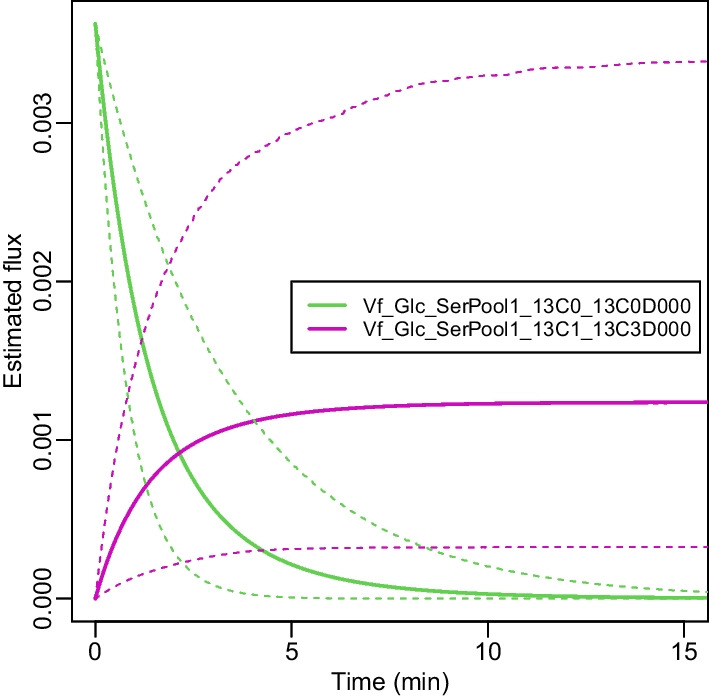
Estimated flux changes over time in lung cancer tissues. Solid curves: estimated fluxes; Dashed curves: 95% credible bounds

We next investigated the posterior mean and standard deviation over time for two fluxes, $Vf_{Glc-SerPool1-13C0-13C0D000}$ and $Vf_{Glc-SerPool1-13C1-13C3D000}$, from the cancer group, as shown in Fig. 3. Based on Fig. 1, we had a non-zero concentration for labeled *Glc* in medium and unlabeled *Glc* in the cytoplasm at time zero. On the one hand, the labeled *Glc* concentration in the cytoplasm increased with time, and the forward flux from labeled *Glc* to labeled *Ser* in cytoplasm also tended to increase. On the other hand, the unlabeled *Glc*’s concentration decreased owing to the activity of forward reactions to other metabolites; similarly the forward flux from unlabeled *Glc* to unlabeled *Ser* in cytoplasm tended to decrease too, as expected for actively metabolizing cells.

**Fig. 4:**
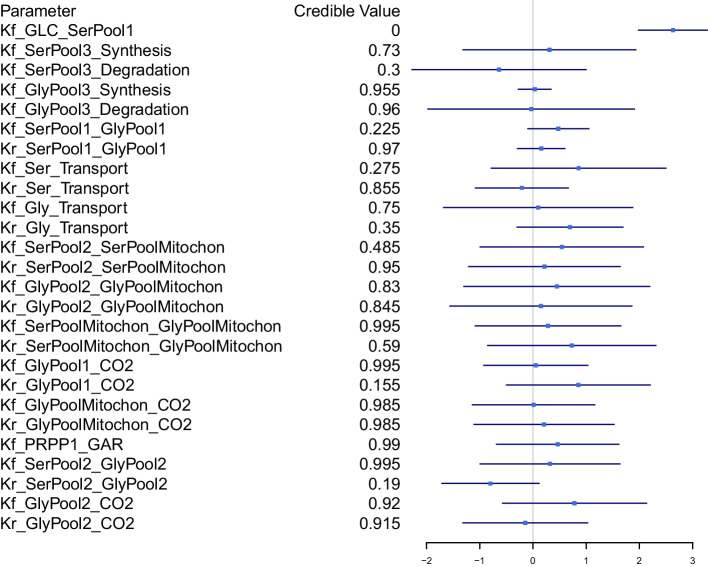
Comparison of each kinetic parameter between lung cancer and non-cancer tissues. The first column lists parameter names, the second column lists credible values and the third column lists 95% credible intervals for the differences in parameter values between cancer and non-cancer tissues

#### Group comparison

We compared each kinetic model parameter between cancer and non-cancer tissues based on our proposed hypothesis testing procedure. Specifically, we calculated the 95% credible interval for the difference in parameter value between cancer and non-cancer tissues and checked whether it covered zero. A credible value was further calculated to quantify the significance level of the test. We obtained the posterior samples based on 2000 MCMC iterations after 5500 burn-in iterations. The convergence of the MCMC chains was examined by the Geweke test (Additional File 2: Table S3). Results are shown in Fig. 4. We found that the forward flux from glucose to serine in pool 1 ($K_{f-GLC-SerPool1}$) is significantly higher in cancer than non-cancer tissues (credible value<0.001). This result is consistent with the result reported in [41], providing evidence for the validity of our method. Further, compared to [41], our method is able to infer the difference in kinetic model parameters in a statistically rigorous way.

**Fig. 5:**
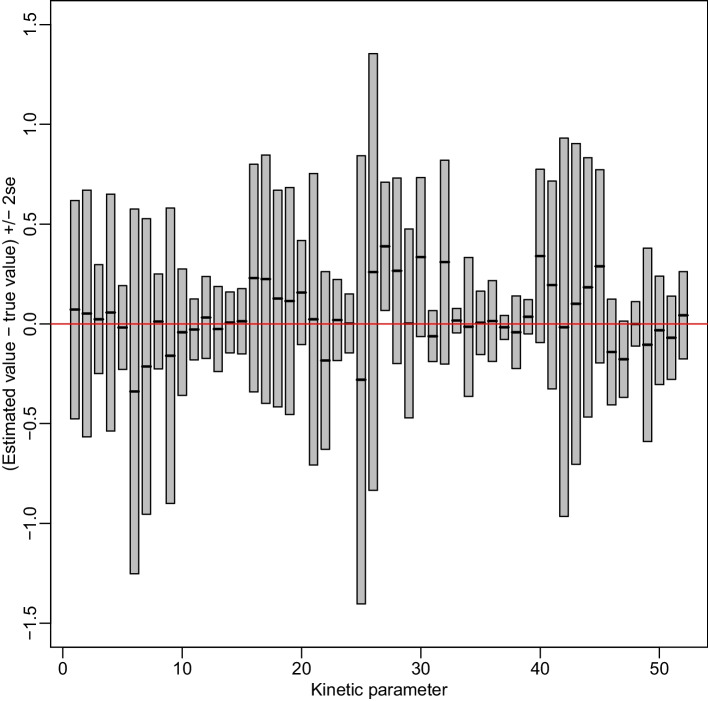
Difference between estimated and true parameter values. Each box presents result for one kinetic parameter. The thick black line in the middle of a box indicates the averaged difference between estimated and true parameter values across 30 simulation runs. The top and bottom of the box indicate averaged difference ± 2 se, respectively. The horizontal red line at zero indicates the target value (no difference betweeen estimated and true values)

### Simulation studies

We conducted simulation studies to evaluate the performance of our method. Data were simulated based on Model (1) with parameters specified according to the real data from [41]. Specifically, we considered the three-pool model, which contains 608 ODEs and 52 parameters, from [41] and borrowed the kinetic parameter estimates from [41] and accordingly set the true values of $\varvec{\beta }$ in our simulations. As to the variances of the assumed normally distributed data errors, we borrowed the variation information in the real data with three replicates in [41] and randomly generated the $\varvec{\sigma }^2$ values from an inverse Gamma distribution which took the shrinkage mean and variance of the real data replicates’ variation as the mean and variance. These randomly generated values were taken as the true values of the data error variances in our simulation studies. Then our simulated concentration data were generated accordingly based on the assumed normal distributed errors.

**Fig. 6:**
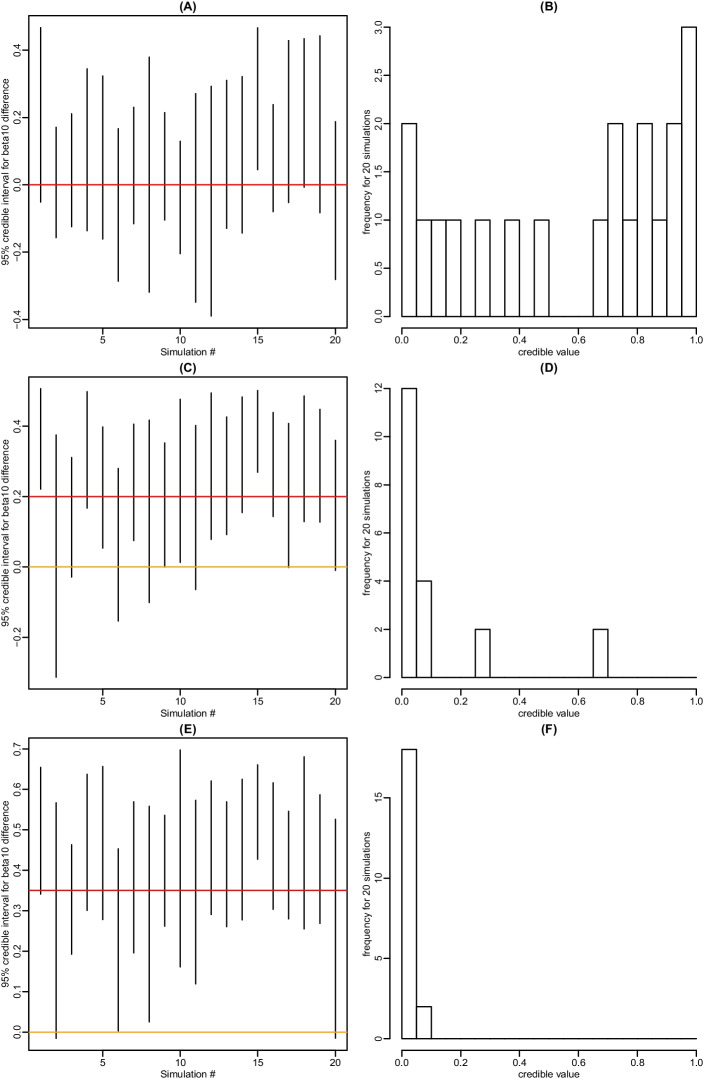
The 95% credible interval of $\beta _{10}^{(1)} - \beta _{10}^{(2)}$ and distribution of the credible value for testing the hypothesis of $\beta _{10}^{(1)} = \beta _{10}^{(2)}$ under the following three scenarios: (A) and (B), $\beta _{10}^{(1)} = \beta _{10}^{(2)}$; (C) and (D), $\beta _{10}^{(1)} - \beta _{10}^{(2)} = 0.2$; and (E) and (F), $\beta _{10}^{(1)} - \beta _{10}^{(2)} = 0.35$. A red line indicates the true value of $\beta _{10}^{(1)} - \beta _{10}^{(2)}$ and an orange line at zero indicates the value of $\beta _{10}^{(1)} - \beta _{10}^{(2)}$ under the null hypothesis. Results are based on 20 simulation runs

#### Parameter estimation

Our first set of simulations evaluated the performance of our proposed parameter estimation method. We simulated data with 3 replicates based on the cancer data from [41]. We applied our method to generate posterior samples from 15,000 MCMC iterations, where the adaptive sampling started after 3000 burn-in iterations and the delayed rejection started after another 1000 iterations. The simulations were repeated 30 times, i.e. 30 seperate datasets were generated. We examined the difference between the estimated parameter value (posterior mean) and true value. Figure 5 plots the averaged difference across 30 simulation runs ± 2 se, indicated by a gray box, for each of the 52 kinetic parameters, where se is the standard error of the difference between estimated and true parameter values across 30 simulation runs. Almost all of the boxes (51 out of 52) covered zero, indicating that the true parameter value was within 2 se of the estimated values. The one exception was $\beta _{27}$, where the true value was outside 2 se. But it was still within 2.5 se of the averaged estimated value. In summary, our estimated values were close to true values relative to the standard errors of the estimated values. Therefore, the simulation results demonstrate that the performance of our estimation method was satisfying.

#### Group comparison

Our second set of simulations assessed the performance of the hypothesis testing method. Data were simulated following the same procedure as the first set of cancer simulations (superscripted by (1)), except that a second non-cancer experimental group (superscripted by (2)) was added. We tested the hypothesis of $\beta _{10}^{(1)} = \beta _{10}^{(2)}$ and considered the following three scenarios: $\beta _{10}^{(1)} = \beta _{10}^{(2)}$, $\beta _{10}^{(2)} = \beta _{10}^{(1)} - 0.2$, and $\beta _{10}^{(2)} = \beta _{10}^{(1)} - 0.35$.

We applied our proposed testing method, which converts the testing problem into one problem of estimating $\eta _{10} = \beta _{10}^{(1)} - \beta _{10}^{(2)}$ and rejects the null hypothesis if the 95% credible interval of $\eta _{10}$ does not cover zero, or equivalently, the credible value is smaller than 0.05. For every simulation, we used 3000 burn-in steps before heading into adaptive phase, and after further 1000 steps, we added delayed rejection. The simulations were repeated 20 times (i.e. 20 datasets were generated) for each scenario and the posterior samples contained 10,000 steps in total. Under the first scenario where the null hypothesis held (Fig. 6A and B), the 95% credible interval covered 0 and thus did not reject the null hypothesis in most of the 20 simulations. The distribution of credible value over 20 simulations was dispersed with many of them having large values, also suggesting the null hypothesis was likely to hold. Under the second scenario where there was a moderate difference between $\beta _{10}^{(1)}$ and $\beta _{10}^{(2)}$ (Fig. 6C and D), the 95% credible interval did not cover 0 in most simulations and the distribution of credible value was very concentrated around small values, both suggesting the null hypothesis was unlikely to hold. Under the third scenario where the true difference was larger (Fig. 6E and F), the 95% credible interval did not cover 0 in almost all simulations and the distribution of credible values all approached to zero value, both strongly suggesting that the null hypothesis should be rejected. In summary, our proposed reparameterization method for hypothesis testing tended to not rejecting the null hypothesis under the scenario that the null hypothesis held, and rejecting the null hypothesis under the scenarios that the null hypothesis did not held. Therefore, these simulation results indicate that our hypothesis testing method was well performed.

## Discussion

Our method has close points of contact with [54], where the authors developed a novel Bayesian approach for the inference of ODEs for characterizing transcription factor activities based on gene expression data. Our method extends this Bayesian idea in multiple ways. First, we propose a new reparameterization method and credible value-based procedure that enables Bayesian hypothesis testing for comparing kinetic parameters between experimental groups. Second, we introduce component-wise adaptive Metropolis and delayed rejection methods to improve the MCMC efficiency for complex ODE model systems with high-dimensional parameters. Third, we propose a Bayesian shrinkage prior to stablize variance parameter estimation under limited sample size.

Our hypothesis testing method for comparing kinetic model parameters can be extended to identify the between-group difference in metabolic flux, which is the net rate of flow of atoms or metabolic subunits through a metabolic pathway or network segment. Based on the kinetic model, the flux can be expressed as a function of *t* and $\varvec{\beta }_k$, where $\varvec{\beta }_k$ is a sub-vector of $\varvec{\beta }$ that are related to the isotopic flow through the $k^{th}$ metabolite. To determine whether the flux stays the same between experimental groups, it is sufficient to evaluate whether $\varvec{\beta }_k^{(1)}$ and $\varvec{\beta }_k^{(2)}$ are equal, where $\varvec{\beta }_k^{(1)}$ and $\varvec{\beta }_k^{(2)}$ are values of $\varvec{\beta }_k$ in the two groups, respectively. Consider the reparameterization that $\varvec{\eta }_k=\varvec{\beta }_k^{(2)}- \varvec{\beta }_k^{(1)}$, the aforementioned hypothesis testing problem becomes $H_0: \varvec{\eta }_k=0 \text { vs. } H_1: \varvec{\eta }_k \ne 0$. We can jointly model data from the two experimental groups and obtain posterior samples of $\varvec{\eta }_k$. The 95% highest density credible region of $\varvec{\eta }_k$ can then be constructed to assess whether the null hypothesis holds. A credible value can also be defined to quantify the level of evidence supporting the null hypothesis. Given the straightforward extension to comparing a vector of parameters, technical difficulties remain in constructing multi-dimensional credible regions. Some successes have been found in the bivariate case [55] or log-concave posterior distributions [56]. However, these algorithms cannot be used in our case since neither the explicit form nor the log-concavity property of the posterior distribution is known. In addition, our current testing strategy is biologically informative because each kinetic parameter in the ODEs is biologically meaningful. The pathway flux can be controlled mainly by one or several enzymes in the pathway, and the ranking of the enzymes by the significance level of their corresponding $\beta$’s helps determine the relative importance of each enzyme in the pathway for flux control (e.g. [40, 57]). This also allows testing of a specific hypothesis that a particular enzyme contributes most to flux control, which would then be a potential therapeutic target.

The credible value approach is easy to implement and practically valid based on simulation studies and real data analysis. Its theoretical performance needs further investigation. We refer to [58] and postulate that the credible value has an asymptotically uniform distribution when the null hypothesis is true, that is, the parameters are identical between cancer and non-cancer group. Further, Xie and Singh [58] demonstrated the asymptotic equivalence of posterior distribution with confidence distribution, which supports our approach of doing hypothesis testing through evaluating the “significance” of null hypothesis under any confidence distribution, and thus through credible value.

An alternative MCMC algorithm, the geometric based sampling methods (mMALA and RMHMC included) [59, 60] may be considered for our model. Those methods, shown to be more efficient than the standard Metropolis-Hastings algorithm and can effectively handle highly correlated samples [59, 60], could be more suitable for applications when the dynamic system presents partially unidentifiable structures commonly seen in biochemical networks [61]. Implementating these methods relies on a fast and accurate solver for the auxiliary sensitivity equations (Eq. (2.1) in [61]) at each sampling iteration, which remains to be explored given the large number of ODE equations and dimension of parameters in our application. This will be our interest as a future research.

## Conclusions

In this paper, we have developed a Bayesian framework for non-steady-state kinetic modeling analysis of SIRM data. By using prior distributions to incorporate biological knowledge and integrate information across metabolites, our method provides robust estimation of model parameters. By introducing a new reparameterization and credible value-based inference procedure, our method allows comparison of kinetic model parameters between experimental groups in a statistically rigorous way. Real data analysis and simulation studies demonstrate that our framework performs well in practical situations.

### Supplementary Information

**Additional file 1**. Supplemental Methods.

**Additional file 2**. Supplemental Tables.

## Data Availability

Our method is freely available for academic use at https://github.com/xuzhang0131/MCMCFlux.

## Abbreviations

SIRM: Stable isotope resolved metabolomics
ODEs: Ordinary differential equations
MCMC: Markov chain Monte Carlo
psd: Posterior standard deviation
sd: Standard deviation
se: Standard error
$\mathbf {^{13}C_6}$-Glc: $^{13}C$-enriched glucose.

## References

[1] Fan TW-M, Lane AN, Higashi RM, Farag MA, Gao H, Bousamra M, Miller DM (2009). Altered regulation of metabolic pathways in human lung cancer discerned by 13C stable isotope-resolved metabolomics (SIRM). Mol Cancer.

[2] Lane AN, Fan TW-M, Xie Z, Moseley HN, Higashi RM (2009). Isotopomer analysis of lipid biosynthesis by high resolution mass spectrometry and NMR. Anal Chim Acta.

[3] Fan TW-M, Lane AN, Higashi RM, Yan J (2011). Stable isotope resolved metabolomics of lung cancer in a SCID mouse model. Metabolomics.

[4] Moseley HN, Lane AN, Belshoff AC, Higashi RM, Fan TW-M (2011). A novel deconvolution method for modeling UDP-N-Acetyl-D-glucosamine biosynthetic pathways based on 13C mass isotopologue profiles under non-steady-state conditions. BMC Biol.

[5] Le A, Lane AN, Hamaker M, Bose S, Gouw A, Barbi J, Tsukamoto T, Rojas CJ, Slusher BS, Zhang H (2012). Glucose-independent glutamine metabolism via TCA cycling for proliferation and survival in B cells. Cell Metab.

[6] Fan TW-M, Tan J, McKinney MM, Lane AN (2012). Stable isotope resolved metabolomics analysis of ribonucleotide and RNA metabolism in human lung cancer cells. Metabolomics.

[7] Fan TW-M, Lorkiewicz PK, Sellers K, Moseley HN, Higashi RM, Lane AN (2012). Stable isotope-resolved metabolomics and applications for drug development. Pharmacol Ther.

[8] Lorkiewicz P, Higashi RM, Lane AN, Fan TW-M (2012). High information throughput analysis of nucleotides and their isotopically enriched isotopologues by direct-infusion FTICR-MS. Metabolomics.

[9] Fan TW-M, Lane AN (2016). Applications of NMR spectroscopy to systems biochemistry. Prog Nucl Magn Reson Spectrosc.

[10] Fan TW-M, Warmoes MO, Sun Q, Song H, Turchan-Cholewo J, Martin JT, Mahan A, Higashi RM, Lane AN (2016). Distinctly perturbed metabolic networks underlie differential tumor tissue damages induced by immune modulator β-glucan in a two-case ex vivo non-small-cell lung cancer study. Molecular Case Studies.

[11] Alarcon-Barrera JC, Kostidis S, Ondo-Mendez A, Giera M (2022). Recent advances in metabolomics analysis for early drug development. Drug Discov Today.

[12] Yuneva MO, Fan TW-M, Allen TD, Higashi RM, Ferraris DV, Tsukamoto T, Matés JM, Alonso FJ, Wang C, Seo Y (2012). The metabolic profile of tumors depends on both the responsible genetic lesion and tissue type. Cell Metab.

[13] Xie H, Hanai J-I, Ren J-G, Kats L, Burgess K, Bhargava P, Signoretti S, Billiard J, Duffy KJ, Grant A, Wang X, Lorkiewicz PK, Schatzman S, Bousamra M, Lane AN, Higashi RM, Fan TWM, Pandolfi PP, Sukhatme VP, Seth P (2014). Targeting lactate dehydrogenase-a inhibits tumorigenesis and tumor progression in mouse models of lung cancer and impacts tumor-initiating cells. Cell Metab..

[14] Sellers K, Fox MP, Bousamra M, Slone SP, Higashi RM, Miller DM, Wang Y, Yan J, Yuneva MO, Deshpande R, Lane AN, Fan TWM (2015). Pyruvate carboxylase is critical for non-small-cell lung cancer proliferation. J Clin Investig..

[15] Fan TW-M, Warmoes MO, Sun Q, Song H, Turchan-Cholewo J, Martin JT, Mahan A, Higashi RM, Lane AN (2016). Distinctly perturbed metabolic networks underlie differential tumor tissue damages induced by immune modulator β-glucan in a two-case ex vivo non-small-cell lung cancer study. Molecular Case Studies.

[16] Jung SM, Le J, Doxsey WG, Haley JA, Park G, Guertin DA, Jang C. Stable isotope tracing and metabolomics to study in vivo brown adipose tissue metabolic fluxes. In: Brown adipose tissue, 2022;119–130. Humana, New York, NY. 10.1007/978-1-0716-2087-8_8 PMC9924221 35167094

[17] Lu D, Mulder H, Zhao P, Burgess SC, Jensen MV, Kamzolova S, Newgard CB, Sherry AD (2002). 13C NMR isotopomer analysis reveals a connection between pyruvate cycling and glucose-stimulated insulin secretion (GSIS). Proc Natl Acad Sci.

[18] DeBerardinis RJ, Mancuso A, Daikhin E, Nissim I, Yudkoff M, Wehrli S, Thompson CB (2007). Beyond aerobic glycolysis: transformed cells can engage in glutamine metabolism that exceeds the requirement for protein and nucleotide synthesis. Proc Natl Acad Sci.

[19] Frezza C, Zheng L, Folger O, Rajagopalan KN, MacKenzie ED, Jerby L, Micaroni M, Chaneton B, Adam J, Hedley A (2011). Haem oxygenase is synthetically lethal with the tumour suppressor fumarate hydratase. Nature.

[20] Mullen AR, Wheaton WW, Jin ES, Chen P-H, Sullivan LB, Cheng T, Yang Y, Linehan WM, Chandel NS, DeBerardinis RJ (2012). Reductive carboxylation supports growth in tumour cells with defective mitochondria. Nature.

[21] Lewis CA, Parker SJ, Fiske BP, McCloskey D, Gui DY, Green CR, Vokes NI, Feist AM, Vander Heiden MG, Metallo CM (2014). Tracing compartmentalized NADPH metabolism in the cytosol and mitochondria of mammalian cells. Mol Cell.

[22] DeNicola GM, Chen P-H, Mullarky E, Sudderth JA, Hu Z, Wu D, Tang H, Xie Y, Asara JM, Huffman KE (2015). NRF2 regulates serine biosynthesis in non-small cell lung cancer. Nat Genet.

[23] Lin P, Dai L, Crooks DR, Neckers LM, Higashi RM, Fan TW, Lane AN (2021). NMR methods for determining lipid turnover via stable isotope resolved metabolomics. Metabolites.

[24] Steuer R, Gross T, Selbig J, Blasius B (2006). Structural kinetic modeling of metabolic networks. Proc Natl Acad Sci.

[25] Jamshidi N, Palsson BØ (2008). Formulating genome-scale kinetic models in the post-genome era. Mol Syst Biol.

[26] Saa PA, Nielsen LK (2016). Construction of feasible and accurate kinetic models of metabolism: a Bayesian approach. Sci Rep.

[27] Saa PA, Nielsen LK (2017). Formulation, construction and analysis of kinetic models of metabolism: A review of modelling frameworks. Biotechnol Adv.

[28] Cuperlovic-Culf M (2018). Machine learning methods for analysis of metabolic data and metabolic pathway modeling. Metabolites.

[29] Schomburg I, Chang A, Hofmann O, Ebeling C, Ehrentreich F, Schomburg D (2002). BRENDA: a resource for enzyme data and metabolic information.

[30] Nöh K, Grönke K, Luo B, Takors R, Oldiges M, Wiechert W (2007). Metabolic flux analysis at ultra short time scale: isotopically non-stationary 13C labeling experiments. J Biotechnol.

[31] Young JD, Walther JL, Antoniewicz MR, Yoo H, Stephanopoulos G (2008). An elementary metabolite unit (EMU) based method of isotopically nonstationary flux analysis. Biotechnol Bioeng.

[32] de Mas IM, Selivanov VA, Marin S, Roca J, Orešič M, Agius L, Cascante M (2011). Compartmentation of glycogen metabolism revealed from 13C isotopologue distributions. BMC Syst Biol.

[33] Wiechert W, Nöh K (2013). Isotopically non-stationary metabolic flux analysis: complex yet highly informative. Curr Opin Biotechnol.

[34] Young JD (2014). INCA: a computational platform for isotopically non-stationary metabolic flux analysis. Bioinformatics.

[35] Foguet C, Marin S, Selivanov VA, Fanchon E, Lee W-NP, Guinovart JJ, de Atauri P, Cascante M (2016). Hepatodyn: a dynamic model of hepatocyte metabolism that integrates 13C isotopomer data. PLoS Comput Biol.

[36] Resat H, Petzold L, Pettigrew MF. In: Ireton R, Montgomery K, Bumgarner R, Samudrala R, McDermott J, editors. Kinetic modeling of biological systems. Totowa: Humana Press; 2009. p. 311–35. 10.1007/978-1-59745-243-4_14 PMC2877599 19381542

[37] Selivanov VA, Vizán P, Mollinedo F, Fan TW-M, Lee PW, Cascante M (2010). Edelfosine-induced metabolic changes in cancer cells that precede the overproduction of reactive oxygen species and apoptosis. BMC Syst Biol.

[38] Cascante M, Selivanov V, Ramos-Montoya A. In: Fan TW-M, Lane AN, Higashi RM, editors. Application of tracer-based metabolomics and flux analysis in targeted cancer drug design. Totowa, NJ: Humana Press; 2012. p. 299–320.

[39] Saa PA, Nielsen LK (2017). Formulation, construction and analysis of kinetic models of metabolism: a review of modelling frameworks. Biotechnol Adv.

[40] Selivanov VA, Marin S, Lee PW, Cascante M (2006). Software for dynamic analysis of tracer-based metabolomic data: estimation of metabolic fluxes and their statistical analysis. Bioinformatics.

[41] Fan TW-M, Bruntz RC, Yang Y, Song H, Chernyavskaya Y, Deng P, Zhang Y, Shah PP, Beverly LJ, Qi Z (2019). De novo synthesis of serine and glycine fuels purine nucleotide biosynthesis in human lung cancer tissues. J Biol Chem.

[42] Haario H, Saksman E, Tamminen J (2005). Componentwise adaptation for high dimensional MCMC. Comput Stat.

[43] Haario H, Saksman E, Tamminen J (2001). An adaptive Metropolis algorithm. Bernoulli.

[44] Haario H, Laine M, Mira A, Saksman E (2006). DRAM: efficient adaptive MCMC. Stat Comput.

[45] Tierney L, Mira A (1999). Some adaptive Monte Carlo methods for Bayesian inference. Stat Med.

[46] Mira A (2001). On Metropolis-Hastings algorithms with delayed rejection. Metron.

[47] Kreutz C, Rodriguez MB, Maiwald T, Seidl M, Blum H, Mohr L, Timmer J (2007). An error model for protein quantification. Bioinformatics.

[48] Chang A, Jeske L, Ulbrich S, Hofmann J, Koblitz J, Schomburg I, Neumann-Schaal M, Jahn D, Schomburg D (2021). Brenda, the elixir core data resource in 2021: new developments and updates. Nucleic Acids Res.

[49] Wu H, Wang C, Wu Z (2013). A new shrinkage estimator for dispersion improves differential expression detection in RNA-seq data. Biostatistics.

[50] Geman S, Geman D (1984). Stochastic relaxation, Gibbs distributions, and the Bayesian restoration of images. IEEE Trans Pattern Anal Mach Intell.

[51] Peskun PH (1973). Optimum Monte-Carlo sampling using Markov chains. Biometrika.

[52] Quan H, Zhang B, Lan Y, Luo X, Chen X (2019). Bayesian hypothesis testing with frequentist characteristics in clinical trials. Contemp Clin Trials.

[53] Wittig U, Rey M, Weidemann A, Kania R, Müller W (2018). SABIO-RK: an updated resource for manually curated biochemical reaction kinetics. Nucleic Acids Res.

[54] Rogers S, Khanin R, Girolami M (2007). Bayesian model-based inference of transcription factor activity. BMC Bioinform.

[55] Turkkan N, Pham-Gia T (1997). Highest posterior density credible region and minimum area confidence region: the bivariate case. Appl Stat.

[56] Pereyra M (2017). Maximum-a-posteriori estimation with Bayesian confidence regions. SIAM J Imag Sci.

[57] Joy MP, Elston TC, Lane AN, Macdonald JM, Cascante M. Introduction to metabolic control analysis (MCA). In: The Handbook of Metabolomics. Springer; 2012. p. 279–97.

[58] Xie M-G, Singh K (2013). Confidence distribution, the frequentist distribution estimator of a parameter: A review. Int Stat Rev.

[59] Girolami M, Calderhead B (2011). Riemann manifold Langevin and Hamiltonian monte Carlo methods. J Royal Stat Soc: Series B (Stat Methodol).

[60] Kramer A, Stathopoulos V, Girolami M, Radde N (2014). MCMC\_CLIB—an advanced MCMC sampling package for ODE models. Bioinformatics.

[61] Calderhead B, Girolami M (2011). Statistical analysis of nonlinear dynamical systems using differential geometric sampling methods. Interface focus.

[62] Hug S, Raue A, Hasenauer J, Bachmann J, Klingmüller U, Timmer J, Theis F (2013). High-dimensional Bayesian parameter estimation: case study for a model of JAK2/STAT5 signaling. Math Biosci.

[63] Sun RC, Fan TW-M, Deng P, Higashi RM, Lane AN, Le A-T, Scott TL, Sun Q, Warmoes MO, Yang Y (2017). Noninvasive liquid diet delivery of stable isotopes into mouse models for deep metabolic network tracing. Nat Commun.

[64] Hanahan D, Weinberg RA (2011). Hallmarks of cancer: the next generation. Cell.

[65] Chen C, Gonzalez FJ, Idle JR (2007). LC-MS-based metabolomics in drug metabolism. Drug Metab Rev.

[66] West FD, Henderson WM, Yu P, Yang J-Y, Stice SL, Smith MA (2012). Metabolomic response of human embryonic stem cell-derived germ-like cells after exposure to steroid hormones. Toxicol Sci.

[67] Kamburov A, Cavill R, Ebbels TM, Herwig R, Keun HC (2011). Integrated pathway-level analysis of transcriptomics and metabolomics data with IMPaLA. Bioinformatics.

[68] Heijne WH, Lamers R-JA, van Bladeren PJ, Groten JP, van Nesselrooij JH, Van Ommen B (2005). Profiles of metabolites and gene expression in rats with chemically induced hepatic necrosis. Toxicol Pathol.

[69] Heijne WH, Jonker D, Stierum RH, van Ommen B, Groten JP (2005). Toxicogenomic analysis of gene expression changes in rat liver after a 28-day oral benzene exposure. Mut Res/Fund Mol Mech Mutagenesis.

[70] Morvan D, Demidem A (2007). Metabolomics by proton nuclear magnetic resonance spectroscopy of the response to chloroethylnitrosourea reveals drug efficacy and tumor adaptive metabolic pathways. Can Res.

[71] Ho JE, Larson MG, Vasan RS, Ghorbani A, Cheng S, Rhee EP, Florez JC, Clish CB, Gerszten RE, Wang TJ (2013). Metabolite profiles during oral glucose challenge. Diabetes.

[72] Kankainen M, Gopalacharyulu P, Holm L, Orešič M (2011). MPEA—-metabolite pathway enrichment analysis. Bioinformatics.

[73] Yoo H, Antoniewicz MR, Stephanopoulos G, Kelleher JK (2008). Quantifying reductive carboxylation flux of glutamine to lipid in a brown adipocyte cell line. J Biol Chem.

[74] Wise DR, Ward PS, Shay JE, Cross JR, Gruber JJ, Sachdeva UM, Platt JM, DeMatteo RG, Simon MC, Thompson CB (2011). Hypoxia promotes isocitrate dehydrogenase-dependent carboxylation of α-ketoglutarate to citrate to support cell growth and viability. Proc Natl Acad Sci.

[75] Metallo CM, Gameiro PA, Bell EL, Mattaini KR, Yang J, Hiller K, Jewell CM, Johnson ZR, Irvine DJ, Guarente L (2012). Reductive glutamine metabolism by IDH1 mediates lipogenesis under hypoxia. Nature.

[76] Yang Y, Lane AN, Ricketts CJ, Sourbier C, Wei M-H, Shuch B, Pike L, Wu M, Rouault TA, Boros LG (2013). Metabolic reprogramming for producing energy and reducing power in fumarate hydratase null cells from hereditary leiomyomatosis renal cell carcinoma. PLoS ONE.

[77] Jiang L, Shestov AA, Swain P, Yang C, Parker SJ, Wang QA, Terada LS, Adams ND, McCabe MT, Pietrak B (2016). Reductive carboxylation supports redox homeostasis during anchorage-independent growth. Nature.

[78] Voit E, Qi Z, Miller G (2008). Steps of modeling complex biological systems. Pharmacopsychiatry.

[79] Qi Z, Miller G, Voit E (2008). A mathematical model of presynaptic dopamine homeostasis: implications for schizophrenia. Pharmacopsychiatry.

[80] Bernardo J, Berger J, Dawid A, Smith A. Efficient metropolis jumping rules. In: Bayesian Statistics, vol. 5. New York: Oxford Univeristy Press; 1996.

[81] Lane AN, Fan TW-M (2015). Regulation of mammalian nucleotide metabolism and biosynthesis. Nucleic Acids Res.

[82] Liu Y-C, Li F, Handler J, Huang CRL, Xiang Y, Neretti N, Sedivy JM, Zeller KI, Dang CV (2008). Global regulation of nucleotide biosynthetic genes by c-Myc. PLoS ONE.

[83] Hori H, Tran P, Carrera CJ, Hori Y, Rosenbach MD, Carson DA, Nobori T (1996). Methylthioadenosine phosphorylase cDNA transfection alters sensitivity to depletion of purine and methionine in A549 lung cancer cells. Can Res.

[84] Hayes JD, Dinkova-Kostova AT (2014). The Nrf2 regulatory network provides an interface between redox and intermediary metabolism. Trends Biochem Sci.

[85] Wikoff WR, Grapov D, Fahrmann JF, DeFelice B, Rom WN, Pass HI, Kim K, Nguyen U, Taylor SL, Gandara DR (2015). Metabolomic markers of altered nucleotide metabolism in early stage adenocarcinoma. Cancer Prev Res.

[86] Tedeschi PM, Vazquez A, Kerrigan JE, Bertino JR (2015). Mitochondrial methylenetetrahydrofolate dehydrogenase (MTHFD2) overexpression is associated with tumor cell proliferation and is a novel target for drug development. Mol Cancer Res.

[87] Maddocks OD, Athineos D, Cheung EC, Lee P, Zhang T, van den Broek NJ, Mackay GM, Labuschagne CF, Gay D, Kruiswijk F (2017). Modulating the therapeutic response of tumours to dietary serine and glycine starvation. Nature.

[88] Paone A, Marani M, Fiascarelli A, Rinaldo S, Giardina G, Contestabile R, Paiardini A, Cutruzzolà F (2014). SHMT1 knockdown induces apoptosis in lung cancer cells by causing uracil misincorporation. Cell Death Dis.

[89] Zhang WC, Shyh-Chang N, Yang H, Rai A, Umashankar S, Ma S, Soh BS, Sun LL, Tai BC, Nga ME (2012). Glycine decarboxylase activity drives non-small cell lung cancer tumor-initiating cells and tumorigenesis. Cell.

[90] Tedeschi PM, Markert EK, Gounder M, Lin H, Dvorzhinski D, Dolfi S, Chan LL, Qiu J, DiPaola R, Hirshfield K (2013). Contribution of serine, folate and glycine metabolism to the ATP, NADPH and purine requirements of cancer cells. Cell Death Dis.

[91] DeNicola GM, Chen P-H, Mullarky E, Sudderth JA, Hu Z, Wu D, Tang H, Xie Y, Asara JM, Huffman KE (2015). NRF2 regulates serine biosynthesis in non-small cell lung cancer. Nat Genet.

[92] Possemato R, Marks KM, Shaul YD, Pacold ME, Kim D, Birsoy K, Sethumadhavan S, Woo H-K, Jang HG, Jha AK (2011). Functional genomics reveal that the serine synthesis pathway is essential in breast cancer. Nature.

[93] Locasale JW, Grassian AR, Melman T, Lyssiotis CA, Mattaini KR, Bass AJ, Heffron G, Metallo CM, Muranen T, Sharfi H (2011). Phosphoglycerate dehydrogenase diverts glycolytic flux and contributes to oncogenesis. Nat Genet.

[94] Schomburg I, Chang A, Schomburg D (2002). BRENDA, enzyme data and metabolic information. Nucleic Acids Res.

[95] Geman S, Geman D (1984). Stochastic relaxation, Gibbs distributions, and the Bayesian restoration of images. IEEE Trans Pattern Anal Mach Intell.

[96] Besag J, York J. Bayesian restoration of images. In: Matsunawa T, editor. Analysis of Statistical Information 1989. p. 491–507.

[97] Gilks WR, Roberts GO, Sahu SK (1998). Adaptive Markov chain Monte Carlo through regeneration. J Am Stat Assoc.

[98] Sahu SK, Zhigljavsky AA (2003). Self-regenerative Markov chain Monte Carlo with adaptation. Bernoulli.

[99] Liebermeister W, Uhlendorf J, Klipp E (2010). Modular rate laws for enzymatic reactions: thermodynamics, elasticities and implementation. Bioinformatics.

[100] Voit EO (2000). Computational analysis of biochemical systems: a practical guide for biochemists and molecular biologists.

[101] Brooks S, Gelman A, Jones G, Meng X-L (2011). Handbook of Markov Chain Monte Carlo.

[102] Kramer A, Stathopoulos V, Girolami M, Radde N (2014). MCMC\_CLIB—an advanced MCMC sampling package for ode models. Bioinformatics.

[103] Akaike H (1974). A new look at the statistical model identification. IEEE Trans Autom Control.

