# Correction: Bayesian kinetic modeling for tracer-based metabolomic data

**DOI:** 10.1186/s12859-024-05635-7

**Published:** 2024-01-10

**Authors:** Xu Zhang, Ya Su, Andrew N. Lane, Arnold J. Stromberg, Teresa W. M. Fan, Chi Wang

**Affiliations:** 1 Dr. Bing Zhang Department of Statistics, University of Kentucky, Lexington, 40536 USA; 2 Department of Statistical Sciences and Operations Research, Virginia Commonwealth University, Richmond, 23220 USA; 3 Markey Cancer Center, University of Kentucky, Lexington, 40536 USA; 4 Center for Environmental and Systems Biochemistry, University of Kentucky, Lexington, 40536 USA; 5 Department of Toxicology and Cancer Biology, University of Kentucky, Lexington, 40536 USA; 6 Division of Cancer Biostatistics, Department of Internal Medicine, University of Kentucky, Lexington, 40536 USA

**Correction to:BMC Bioinformatics (2023) 24:108** 10.1186/s12859-023-05211-5

Following the publication of the original article [1], the authors identified that the funding note was incorrect. The correct funding note is given below.

The incorrect funding is:

This work was supported by National Institutes of Health [1R03CA211835, 5P20GM103436-15, 1P01CA163223-01A1], the Biostatistics and Bioinformatics and Redox Metabolism Shared Resource Facilities of the University of Kentucky Markey Cancer Center [P30CA177558] and University of Kentucky Center for Cancer and Metabolism [1P20GM121327-01].

The correct funding is:

This work was supported by National Institutes of Health [1R03CA211835, 5P20GM103436-15, 1P01CA163223-01A1], the Biostatistics and Bioinformatics and Redox Metabolism Shared Resource Facilities of the University of Kentucky Markey Cancer Center [P30CA177558] and University of Kentucky Center for Cancer and Metabolism [P20GM121327].

The original article [1] has been corrected.
