# Supplementary material for: Bayesian kinetic modeling for tracer-based metabolomic data
Source: BMC Bioinformatics. 2023 Mar 22;24:108. doi: 10.1186/s12859-023-05211-5 (PMC10035190; doi:10.1186/s12859-023-05211-5)
Supplement: Supplementary file 1 — Additional file 1. Supplemental Methods. [file 12859_2023_5211_MOESM1_ESM.pdf]

Supplemental Methods

S1 Posterior samplng of kinetic parameters

To estimate kinetic parameters, we used component-wise adaptive Metropolis algorithm with delayed rejection (Haario *et al.*, 2005-Haario *et al.*, 2006). Under the adaptive Metropolis algorithm, at the $(l+1)^{th}$ iteration, the proposal distribution $q(\cdot)$ is

$$q(\boldsymbol{\beta}^*|\boldsymbol{\beta}^l) = MVN(\boldsymbol{\beta}^l, C^l)$$

where

$$C^l = \begin{cases} C_0, & l \leq l_0 \\ s_d cov(\boldsymbol{\beta}^1, \ldots, \boldsymbol{\beta}^l) + s_d \epsilon I_d, & l > l_0 \end{cases}$$

and we used the one-dimension conditional version of the $q(\cdot)$. As suggested in the *remark* 1 and 2 of Haario *et al.* (2001), we chose $s_d = \frac{2.4^2}{d}$ (d is the dimension of $\boldsymbol{\beta}$) for practical computation. We also chose each element of C_0 equal to s_d/d, following the code of Hug *et al.* (2013). In adidtion, we computationally reduced the possibility of generating a singular covariance matrix by setting $\epsilon = 1e^{-6}$, which is essential to our adaptive algorithm stated below. To further improve the chain's mixing structure, we also adopted the delayed rejection method (Tierney and Mira, 1999; Mira *et al.*, 2001).

Algorithm S1.1 *Component-wise adaptive Metropolis algorithm with delayed rejection to sample points for the parameter vector* $\boldsymbol{\beta}$.

1. *Choose starting values for* $\boldsymbol{\beta}$*, the sample size* N *and fix the error term variances* σ_i^2.
2. *At sample step* $l+1$*, **for*** $i = 1 : d$*, **do***
   (a) *Propose* β_i^* *from*

$$\begin{aligned} q(\beta_i^*|\beta_i^l) &= q(\beta_i^*|\beta_i^l; \beta_1^{l+1}, \ldots, \beta_{i-1}^{l+1}, \beta_{i+1}^l, \ldots, \beta_d^l) \\ &= N(\beta_i^l, C_{ii}^l - C_{i,-i}^l {C_{-i-i}^l}^{-1} C_{-i,i}^l) \end{aligned}$$

$$C^l = \begin{bmatrix} C_{11}^l & C_{12}^l & \ldots & C_{1d}^l \\ C_{21}^l & C_{22}^l & \ldots & C_{2d}^l \\ \vdots & \vdots & \ddots & \vdots \\ C_{d1}^l & C_{d2}^l & \ldots & C_{dd}^l \end{bmatrix}$$

$$C_{-i-i}^l = \begin{bmatrix} C_{11}^l & \ldots & C_{1,i-1}^l & C_{1,i+1}^l & \ldots & C_{1d}^l \\ \vdots & \vdots & \vdots & \vdots & \vdots & \vdots \\ C_{i-1,1}^l & \ldots & C_{i-1,i-1}^l & C_{i-1,i+1}^l & \ldots & C_{i-1,d}^l \\ C_{i+1,1}^l & \ldots & C_{i+1,i-1}^l & C_{i+1,i+1}^l & \ldots & C_{i+1,d}^l \\ \vdots & \vdots & \vdots & \vdots & \vdots & \vdots \\ C_{d1}^l & \ldots & C_{d,i-1}^l & C_{d,i+1}^l & \ldots & C_{dd}^l \end{bmatrix}$$

$$C^l_{i,-i} = (C^l_{i1}, \dots, C^l_{i,i-1}, C^l_{i,i+1}, \dots, C^l_{i,d})$$
$$C^l_{-i,i} = (C^l_{1i}, \dots, C^l_{i-1,i}, C^l_{i+1,i}, \dots, C^l_{d,i})^T$$

And respectively,

$$q(\beta^l_i|\beta^*_i) = q(\beta^l_i|\beta^*_i; \beta^{l+1}_1, \dots, \beta^{l+1}_{i-1}, \beta^l_{i+1}, \dots, \beta^l_d)$$
$$= N(\beta^*_i, C^l_{ii} - C^l_{i,-i} {C^l_{-i-i}}^{-1} C^l_{-i,i})$$

(b) *Draw some number a randomly from* $N(0,1)$*, then update* β^{l+1}_i *by*

$$\beta^{l+1}_i = \begin{cases} \beta^l_i, & a > \alpha(\beta^l_i, \beta^*_i) \\ \beta^*_i, & a \leq \alpha(\beta^l_i, \beta^*_i) \end{cases}$$

where

$$\alpha(\beta^l_i, \beta^*_i) = \min\{1, \frac{P(\beta^*_i|\log \boldsymbol{y}, \boldsymbol{\beta}_{-i}) q(\beta^l_i|\beta^*_i)}{P(\beta^l_i|\log \boldsymbol{y}, \boldsymbol{\beta}_{-i}) q(\beta^*_i|\beta^l_i)}\}$$
$$= \min\{1, \frac{P(\beta^*_i|\log \boldsymbol{y}, \boldsymbol{\beta}_{-i})}{P(\beta^l_i|\log \boldsymbol{y}, \boldsymbol{\beta}_{-i})}\}$$

and

$$\boldsymbol{\beta}_{-i} = (\beta^{l+1}_1, \dots, \beta^{l+1}_{i-1}, \beta^l_{i+1}, \dots, \beta^l_d).$$

(c) *Delayed rejection step: when the above step rejects* β^*_i*, we'll do a further Metropolis step with scaled covariance.*

i. *Propose another* β^{**}_i *from*

$$q'(\beta^{**}_i|\beta^l_i, \beta^*_i) = N(\mu = \beta^l_i, \sigma^2 = \gamma(C^l_{ii} - C^l_{i,-i} {C^l_{-i-i}}^{-1} C^l_{-i,i}))$$

where γ *is the scale parameter for delayed rejection.*

ii. *Draw a number* a^* *randomly from* $N(0,1)$*, then update* β^{l+1}_i *by*

$$\beta^{l+1}_i = \begin{cases} \beta^l_i, & a^* > \alpha(\beta^l_i, \beta^*_i, \beta^{**}_i) \\ \beta^{**}_i, & a^* \leq \alpha(\beta^l_i, \beta^*_i, \beta^{**}_i) \end{cases}$$

where

$$\alpha(\beta^l_i, \beta^*_i, \beta^{**}_i) = \min\{1,$$
$$\frac{P(\beta^{**}_i|\log \boldsymbol{y}, \boldsymbol{\beta}_{-i}) q(\beta^*_i|\beta^{**}_i)[1 - \alpha(\beta^{**}_i, \beta^*_i)]}{P(\beta^l_i|\log \boldsymbol{y}, \boldsymbol{\beta}_{-i}) q(\beta^*_i|\beta^l_i)[1 - \alpha(\beta^l_i, \beta^*_i)]}\}$$

and

$$q(\beta^*_i|\beta^{**}_i) = N(\beta^*_i, \mu = \beta^{**}_i, \sigma^2 = C^l_{ii} - C^l_{i,-i} {C^l_{-i-i}}^{-1} C^l_{-i,i})$$
$$q(\beta^{**}_i|\beta^*_i) = N(\beta^{**}_i, \mu = \beta^*_i, \sigma^2 = C^l_{ii} - C^l_{i,-i} {C^l_{-i-i}}^{-1} C^l_{-i,i})$$
$$\alpha(\beta^{**}_i, \beta^*_i) = \min\{1, \frac{P(\beta^*_i|\log \boldsymbol{y}, \boldsymbol{\beta}_{-i}) q(\beta^{**}_i|\beta^*_i)}{P(\beta^{**}_i|\log \boldsymbol{y}, \boldsymbol{\beta}_{-i}) q(\beta^*_i|\beta^{**}_i)}\}$$
$$q'(\beta^l_i|\beta^{**}_i, \beta^*_i) = N(\beta^l_i, \mu = \beta^{**}_i, \sigma^2 = \gamma(C^l_{ii} - C^l_{i,-i} {C^l_{-i-i}}^{-1} C^l_{-i,i}))$$

end.

3. *Repeat* ***Step 2*** *until* N *samples are drawn.*

S2 Posterior sampling of error variances

Denote $\boldsymbol{y}_{obs} = (y_1, \ldots, y_n)'$ as the observed concentrations of n isotopomers at one time point. Since we assumed the metabolites' concentrations are independent at the same time point, the conditional distribution of σ_i^2 is only related to $\boldsymbol{y}_{obs,i}$ and the kinetic parameters $\boldsymbol{\beta}$, then we can see that the inverse gamma prior distribution for σ_i^2 is a conjugate prior, which means the conditional posterior distribution of σ_i^2 given $\boldsymbol{y}_{obs}$ and $\boldsymbol{\beta}$ is also in the family of inverse gamma distribution. Thus, when the prior assumption of σ_i^2 has the following distribution

$$p(\sigma_i^2) = \frac{\kappa^\alpha}{\Gamma(\alpha)}(\sigma_i^2)^{-\alpha-1}e^{-\frac{\kappa}{\sigma_i^2}}$$

and the observations $\boldsymbol{y}_{obs}$ follows a normal distribution

$$p(\log \boldsymbol{y}_{obs}|\boldsymbol{\beta}, \Sigma) = \prod_i \frac{1}{\sqrt{2\pi\sigma_i^2}} e^{-\frac{(\log \boldsymbol{y}_{obs,i} - \log \mu_i)^2}{2\sigma_i^2}}$$

then the conditional posterior distribution of σ_i^2 is

$$p(\sigma_i^2|\log \boldsymbol{y}_{obs}, \boldsymbol{\beta}, \sigma_{j\neq i}^2) \propto (\sigma_i^2)^{-\alpha-1-\frac{1}{2}} e^{-\frac{1}{\sigma_i^2}(\kappa+\frac{(\log \boldsymbol{y}_{obs,i}-\log \mu_i)^2}{2})}$$

When we have concentration data for T time points and m independent replicate observations for each isotopomer at one time point, and $\boldsymbol{y} = (\boldsymbol{y}_{tj})$ is the observed data $(t = 1, \ldots, T; j = 1, \ldots, m)$, the conditional posterior distribution of σ_i^2 is

$$p(\sigma_i^2|\log \boldsymbol{y}, \boldsymbol{\beta}, \sigma_{j\neq i}^2) \propto (\sigma_i^2)^{-\alpha-1-\frac{mT}{2}} e^{-\frac{1}{\sigma_i^2}(\kappa+\frac{\sum_{t=1}^T\sum_{j=1}^m(\log y_{tji}-\log \mu_{ti})^2}{2})}.$$

References

Haario, H., Saksman, E., Tamminen, J., *et al.* (2001). An adaptive Metropolis algorithm. *Bernoulli*, **7**(2), 223–242.

Haario, H., Saksman, E., and Tamminen, J. (2005). Componentwise adaptation for high dimensional MCMC. *Computational Statistics*, **20**(2), 265–273.

Haario, H., Laine, M., Mira, A., and Saksman, E. (2006). DRAM: efficient adaptive MCMC. *Statistics and computing*, **16**(4), 339–354.

Hug, S., Raue, A., Hasenauer, J., Bachmann, J., Klingmüller, U., Timmer, J., and Theis, F. (2013). High-dimensional bayesian parameter estimation: Case study for a model of JAK2/STAT5 signaling. *Mathematical Biosciences*, **246**(2), 293–304.

Mira, A. *et al.* (2001). On Metropolis-Hastings algorithms with delayed rejection. *Metron*, **59**(3-4), 231–241.

Tierney, L. and Mira, A. (1999). Some adaptive Monte Carlo methods for Bayesian inference. *Statistics in medicine*, **18**(17-18), 2507–2515.
