# Supplementary material for: Bayesian kinetic modeling for tracer-based metabolomic data
Source: BMC Bioinformatics. 2023 Mar 22;24:108. doi: 10.1186/s12859-023-05211-5 (PMC10035190; doi:10.1186/s12859-023-05211-5)
Supplement: Supplementary file 2 — Additional file 2. Supplemental Tables. [file 12859_2023_5211_MOESM2_ESM.pdf]

Supplemental Tables

Table S1: Prior ranges of kinetic parameters used in the Real Data Analysis section.

Kinetic Parameter	Lower Bound	Upper Bound
Kf_GLC_Transport	0.00	0.01
Kr_GLC_Transport	0.10	1.00
Kf_GLC_SerPool1	0.00	0.10
Kf_GLC_PYR	0.10	1.00
Kf_GLC_PRPP	0.00	0.01
Kf_SerPool3_Synthesis	0.00	0.00
Kf_SerPool3_Degradation	0.00	1.00
Kf_GlyPool3_Synthesis	0.00	0.00
Kf_GlyPool3_Degradation	0.00	0.02
Kf_SerPool1_GlyPool1	50.00	500.00
Kr_SerPool1_GlyPool1	1.00	10.00
Kf_Ser_Transport	0.00	0.01
Kr_Ser_Transport	0.00	0.10
Kf_Gly_Transport	0.00	0.00
Kr_Gly_Transport	0.00	0.02
Kf_SerPool2_SerPoolMitochon	0.00	0.02
Kr_SerPool2_SerPoolMitochon	0.00	0.03
Kf_GlyPool2_GlyPoolMitochon	0.00	0.01
Kr_GlyPool2_GlyPoolMitochon	0.00	0.05
Kf_SerPoolMitochon_GlyPoolMitochon	100.00	1000.00
Kr_SerPoolMitochon_GlyPoolMitochon	1.00	20.00
Kf_GlyPool1_CO2	10.00	100.00
Kr_GlyPool1_CO2	0.00	2.00
Kf_MethyleneTHF_FormylTHF_Cytoplasm	0.00	3.00
Kr_MethyleneTHF_FormylTHF_Cytoplasm	0.10	10.00
Kf_FormylTHF_Formate_Cytoplasm	0.00	3.00
Kr_FormylTHF_Formate_Cytoplasm	200000.00	10000000.00
Kf_GlyPoolMitochon_CO2	100.00	1000.00
Kr_GlyPoolMitochon_CO2	0.10	4.00
Kf_MethyleneTHF_FormylTHF_Mitochon	0.20	10.00
Kr_MethyleneTHF_FormylTHF_Mitochon	0.02	1.00
Kf_FormylTHF_Formate_Mitochon	0.20	10.00
Kr_FormylTHF_Formate_Mitochon	10000.00	100000.00
Kf_PRPP1_GAR	10.00	100.00
Kf_GAR_FGAR	20000.00	200000.00
Kf_FGAR_AMP	20000.00	200000.00
Kf_IMP_AMP	0.00	0.25
Kf_AMP_Degradation	0.00	0.01
Kr_MethyleneTHF_Transport	0.00	0.50
Kf_MethyleneTHF_Transport	0.00	0.30
Kr_FormylTHF_Transport	1.00	50.00
Kf_FormylTHF_Transport	0.05	5.00
Kr_THF_Transport	0.10	8.00
Kf_THF_Transport	0.01	1.00
Kr_Formate_Transport	0.50	10.00
Kf_Formate_Transport	0.10	10.00
Kf_PRPP2_GAR	5.00	100.00
Kf_PRPP3_GAR	1.00	50.00
Kf_SerPool2_GlyPool2	50.00	500.00
Kr_SerPool2_GlyPool2	1.00	500.00
Kf_GlyPool2_CO2	1.00	100.00
Kr_GlyPool2_CO2	0.00	3.00

Table S2: Geweke test results for checking convergence of MCMC chains for estimating kinetic parameters in cancer and non-cancer tissue samples in the Real Data Analysis section.

	Adjusted p-value	
Kinetic Parameter	Cancer	Non-cancer
Kf_GLC_Transport	1.00	1.00
Kr_GLC_Transport	1.00	1.00
Kf_GLC_SerPool1	1.00	0.37
Kf_GLC_PYR	1.00	1.00
Kf_GLC_PRPP	1.00	1.00
Kf_SerPool3_Synthesis	1.00	1.00
Kf_SerPool3_Degradation	1.00	1.00
Kf_GlyPool3_Synthesis	1.00	1.00
Kf_GlyPool3_Degradation	1.00	0.45
Kf_SerPool1_GlyPool1	1.00	1.00
Kr_SerPool1_GlyPool1	1.00	0.09
Kf_Ser_Transport	1.00	1.00
Kr_Ser_Transport	1.00	1.00
Kf_Gly_Transport	1.00	0.03
Kr_Gly_Transport	1.00	1.00
Kf_SerPool2_SerPoolMitochon	1.00	1.00
Kr_SerPool2_SerPoolMitochon	1.00	1.00
Kf_GlyPool2_GlyPoolMitochon	1.00	1.00
Kr_GlyPool2_GlyPoolMitochon	1.00	1.00
Kf_SerPoolMitochon_GlyPoolMitochon	1.00	1.00
Kr_SerPoolMitochon_GlyPoolMitochon	1.00	1.00
Kf_GlyPool1_CO2	1.00	1.00
Kr_GlyPool1_CO2	1.00	1.00
Kf_MethyleneTHF_FormylTHF_Cytoplasm	1.00	1.00
Kr_MethyleneTHF_FormylTHF_Cytoplasm	1.00	0.05
Kf_FormylTHF_Formate_Cytoplasm	0.60	1.00
Kr_FormylTHF_Formate_Cytoplasm	1.00	0.10
Kf_GlyPoolMitochon_CO2	1.00	1.00
Kr_GlyPoolMitochon_CO2	1.00	1.00
Kf_MethyleneTHF_FormylTHF_Mitochon	1.00	1.00
Kr_MethyleneTHF_FormylTHF_Mitochon	1.00	1.00
Kf_FormylTHF_Formate_Mitochon	1.00	1.00
Kr_FormylTHF_Formate_Mitochon	1.00	1.00
Kf_PRPP1_GAR	1.00	1.00
Kf_GAR_FGAR	1.00	1.00
Kf_FGAR_AMP	0.85	1.00
Kf_IMP_AMP	1.00	1.00
Kf_AMP_Degradation	1.00	1.00
Kr_MethyleneTHF_Transport	1.00	1.00
Kf_MethyleneTHF_Transport	1.00	1.00
Kr_FormylTHF_Transport	1.00	1.00
Kf_FormylTHF_Transport	1.00	0.58
Kr_THF_Transport	1.00	1.00
Kf_THF_Transport	1.00	1.00
Kr_Formate_Transport	1.00	1.00
Kf_Formate_Transport	1.00	1.00
Kf_PRPP2_GAR	0.15	1.00
Kf_PRPP3_GAR	1.00	1.00
Kf_SerPool2_GlyPool2	0.70	1.00
Kr_SerPool2_GlyPool2	1.00	1.00
Kf_GlyPool2_CO2	1.00	1.00
Kr_GlyPool2_CO2	1.00	1.00

Note: the p-value adjustment was performed based on the Bonferroni correction for multiple comparisons.

Table S3: Geweke test results for checking convergence of MCMC chains for comparing model parameters between cancer and non-cancer tissues in the Real Data Analysis section.

Kinetic Parameter	Adjusted p-value
Kf_GLC_SerPool1	1.00
Kf_SerPool3_Synthesis	1.00
Kf_SerPool3_Degradation	1.00
Kf_GlyPool3_Synthesis	1.00
Kf_GlyPool3_Degradation	1.00
Kf_SerPool1_GlyPool1	1.00
Kr_SerPool1_GlyPool1	1.00
Kf_Ser_Transport	1.00
Kr_Ser_Transport	1.00
Kf_Gly_Transport	1.00
Kr_Gly_Transport	1.00
Kf_SerPool2_SerPoolMitochon	1.00
Kr_SerPool2_SerPoolMitochon	1.00
Kf_GlyPool2_GlyPoolMitochon	1.00
Kr_GlyPool2_GlyPoolMitochon	1.00
Kf_SerPoolMitochon_GlyPoolMitochon	1.00
Kr_SerPoolMitochon_GlyPoolMitochon	1.00
Kf_GlyPool1_CO2	1.00
Kr_GlyPool1_CO2	1.00
Kf_GlyPoolMitochon_CO2	1.00
Kr_GlyPoolMitochon_CO2	1.00
Kf_PRPP1_GAR	1.00
Kf_SerPool2_GlyPool2	1.00
Kr_SerPool2_GlyPool2	1.00
Kf_GlyPool2_CO2	1.00
Kr_GlyPool2_CO2	1.00

Note: the p-value adjustment was performed based on the Bonferroni correction for multiple comparisons.
